# Photoacoustic Imaging of Human Vasculature Using LED versus Laser Illumination: A Comparison Study on Tissue Phantoms and In Vivo Humans

**DOI:** 10.3390/s21020424

**Published:** 2021-01-09

**Authors:** Sumit Agrawal, Mithun Kuniyil Ajith Singh, Kerrick Johnstonbaugh, David C. Han, Colette R. Pameijer, Sri-Rajasekhar Kothapalli

**Affiliations:** 1 Department of Biomedical Engineering, Pennsylvania State University, University Park, State College, PA 16802, USA; sua347@psu.edu (S.A.); kjohnstonbaugh97@gmail.com (K.J.); 2 Research & Business Development Division, CYBERDYNE INC, Cambridge Innovation Center, 3013 AK Rotterdam, The Netherlands; mithun_ajith@cyberdyne.jp; 3 Department of Surgery, Penn State Heart and Vascular Institute, Hershey, PA 16802, USA; dch15@psu.edu; 4 Penn State Hershey College of Medicine and Milton S. Hershey Medical Center, Hershey, PA 17033, USA; cpameijer@pennstatehealth.psu.edu; 5 Penn State Cancer Institute, Pennsylvania State University, Hershey, PA 17033, USA; 6 Graduate Program in Acoustics, Pennsylvania State University, University Park, State College, PA 16802, USA

**Keywords:** deep tissue imaging, hemangioma, laser, light-emitting diodes (LED), mobile health, peripheral arterial disease, photoacoustic imaging, stroke, vascular malformations

## Abstract

Vascular diseases are becoming an epidemic with an increasing aging population and increases in obesity and type II diabetes. Point-of-care (POC) diagnosis and monitoring of vascular diseases is an unmet medical need. Photoacoustic imaging (PAI) provides label-free multiparametric information of deep vasculature based on strong absorption of light photons by hemoglobin molecules. However, conventional PAI systems use bulky nanosecond lasers which hinders POC applications. Recently, light-emitting diodes (LEDs) have emerged as cost-effective and portable optical sources for the PAI of living subjects. However, state-of-art LED arrays carry significantly lower optical energy (<0.5 mJ/pulse) and high pulse repetition frequencies (PRFs) (4 KHz) compared to the high-power laser sources (100 mJ/pulse) with low PRFs of 10 Hz. Given these tradeoffs between portability, cost, optical energy and frame rate, this work systematically studies the deep tissue PAI performance of LED and laser illuminations to help select a suitable source for a given biomedical application. To draw a fair comparison, we developed a fiberoptic array that delivers laser illumination similar to the LED array and uses the same ultrasound transducer and data acquisition platform for PAI with these two illuminations. Several controlled studies on tissue phantoms demonstrated that portable LED arrays with high frame averaging show higher signal-to-noise ratios (SNRs) of up to 30 mm depth, and the high-energy laser source was found to be more effective for imaging depths greater than 30 mm at similar frame rates. Label-free in vivo imaging of human hand vasculature studies further confirmed that the vascular contrast from LED-PAI is similar to laser-PAI for up to 2 cm depths. Therefore, LED-PAI systems have strong potential to be a mobile health care technology for diagnosing vascular diseases such as peripheral arterial disease and stroke in POC and resource poor settings.

## 1. Introduction

Vascular diseases are the leading cause of death worldwide. Some common vascular diseases include cardiovascular disease, stroke and peripheral artery disease (PAD) [1,2,3]. Many of these vascular diseases need point-of-care (POC) diagnosis and monitoring using nonionizing, noninvasive and cost-effective approaches. Although Doppler ultrasound meets all these requirements, it only maps blood flow, which is operator dependent and influenced by motion artifacts, resulting in limited sensitivity and specificity to detect the disease in its early stage [4]. A POC technique that provides direct label-free molecular and functional information of vasculature is needed to reliably detect and monitor vascular diseases [5,6].

Photoacoustic imaging (PAI) is a hybrid imaging modality that provides rich optical spectroscopic contrast at ultrasonic penetration depths and resolutions [7]. Over the past two decades, PAI has emerged as a promising tool for label-free imaging of individual blood vessels [8,9], detection of angiogenesis [10] and has also helped in extracting several physiologically relevant parameters such as blood oxygen saturation [11,12] and changes in the blood volume [13], which are vital for monitoring disease progression [14,15]. The basic mechanism of PAI includes a nanosecond pulsed optical excitation that illuminates the biological subject. The light absorbing molecules inside the tissue undergo thermoelastic expansion and generate broadband acoustic waves which are subsequently detected using conventional ultrasound (US) detectors [7]. Biomedical PAI mainly capitalizes on the intrinsic absorbers present in human tissue [8], such as oxy- and deoxyhemoglobin [10,11], melanin [16], lipids [17,18], water [19], RNA and DNA [20], as each of these exhibits a characteristic absorption spectrum. However, if the spectral contrast from these intrinsic chromophores is not sufficient to reveal the disease, a wide range of extrinsic contrast agents [21,22,23,24,25] can be functionalized to target different diseased biomarkers to increase molecular sensitivity and specificity.

While ultrasound and certain optical technologies are available in the size of a mobile phone [26,27], PAI systems still have to reach that level of portability. Conventional PAI systems employ bulky class-IV laser sources (100 mJ/pulse) and data acquisition systems to increase the peak imaging performance [28,29,30,31]. Such lasers not only increase the system cost and footprint but also carry a high risk of class-IV exposure. However, to translate PAI technology to POC clinical applications and to resource-limited settings, a significant reduction in both cost and size is required. To address this challenge, several cost-effective alternatives for both the optical excitation [32,33,34,35] and the ultrasound detection [36,37,38,39] components have been explored, including for wearable applications [40,41].

Recently, low-power laser diodes [42,43] and light-emitting-diodes (LEDs) [44] have been proposed as alternatives to laser sources. Specifically, pertaining to their noncoherent nature, LEDs carry a huge potential to be the safe and cost-effective alternative illumination sources for PAI [43,44]. However, compared to the class-IV lasers, LED arrays carry much less optical energy (i.e., order of 100′s μJ) per pulse, and thus their PAI capabilities as a function of imaging depth need to be studied in detail to employ a suitable optical source for a given POC application [45,46].

To enhance the performance of LEDs, an arrayed arrangement of LED elements was developed [47,48], thereby increasing the pulse energies from a few μJ to hundreds of μJ. In addition to this, higher pulse repetition frequency (PRF) rates (i.e., ~4 KHz) of the LEDs allowed a sufficient PA frame averaging which led to significant signal-to-noise ratio (SNR) improvements for deep tissue targets [46]. These LED array B-mode PAUS [47,48] and tomographic imaging [49,50] setups have been demonstrated using several preclinical small animals [51,52] and in vivo human imaging studies [53,54,55].

To date, there is no study that quantitatively compares the PAI performance of LEDs and laser illumination head-to-head. Given these tradeoffs between portability, cost, optical energy and frame rate, this work systematically studies the deep tissue PAI performance of LED and laser excitation to help select a suitable source for a given biomedical application. First, a setup for sequentially performing PAI with these two optical illuminations has been developed. Controlled studies on different tissue phantoms have been performed for detailed evaluation of the imaging performance. Further, in vivo human hand vasculature imaging in 2-D and 3-D was performed using these two optical sources. The rest of the paper is organized as follows. Section 2 describes our proposed setup for comparing the two sources. Comparison studies including tissue-mimicking phantoms and the in vivo human imaging are presented in Section 3. Section 4 provides a detailed discussion of the results.

## 2. Materials and Methods

In this section, a detailed description of the experimental setup for studying the PAI capabilities of high-power laser and LED array sources is presented. First, the commercial LED array-based B-mode PA and ultrasound (US) imaging system (AcousticX, Cyberdyne Inc., Ibaraki, Japan), referred to in this paper as LED-PAUS, is presented in Section 2.1 and then the modifications performed to use the same AcousticX data acquisition system for laser-illumination-based PA, referred to as laser-PAUS, without interrupting parameters of the imaging system, are presented in Section 2.2.

### 2.1. LED Array-Based US/PA (LED-PAUS) Imaging System Description

**Figure 1:**
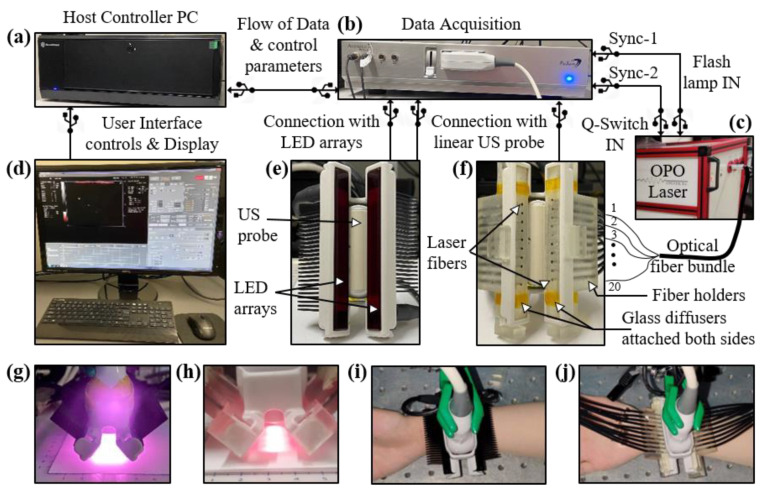
Description of the experimental setup designed for comparing light-emitting diode (LED)-based and high-power laser-based photoacoustic (PA) and ultrasound (US) imaging. The setup consists of the following key components: Commercial B-mode LED-PAUS system (AcousticX, Cyberdyne Inc., Ibaraki, Japan) with (**a**) a host controller PC and (**b**) data acquisition hardware. (**c**) A portable high-power laser (Phocus Mobile, Opotek Inc., Carlsbad, CA, USA) with its output coupled to the input end of an optical fiber bundle. The fiber bundle is split into twenty smaller fibers at the distal end. (**d**) Computer display: displays B-mode US (grayscale), PA (red scale), and coregistered US + PA (overlaid red PA on gray US). The interface also enables switching between LED and laser operation. (**e**) Arrangement of two 850 nm LED arrays around the US probe. (**f**) Arrangement of twenty laser fibers inserted into the two fiber holders around the US probe. Two glass diffusers attached at the fiber output ends to provide uniform laser illumination on the tissue surface. Optical illumination profile achieved with (**g**) two LED array sources and (**h**) laser source. (**i**,**j**) Pictures of a human wrist under imaging with the LED and laser arrangements, respectively.

Conventionally, a high-power and bulky laser source is employed for most B-mode PA and US systems [28,29]. The commercial LED-PAUS system, as shown in Figure 1, consists of a host controller (Figure 1a), data acquisition hardware (Figure 1b) and an interactive graphical user interface shown on the display in Figure 1d. Here, a linear ultrasound probe is sandwiched between two LED arrays to capture interleaved B-mode PA and US images, as shown in Figure 1e. Each LED array consists of four rows of 36 LED elements (1 × 1 mm size). The LED arrays used in this study consist of 850 nm LED elements and each array provides an output energy of 200 μJ/pulse. The excitation pulse widths for each of these LED arrays can be controlled with the software in the range of 30 to 150 ns. For all the experiments in this study, a pulse width of 70 ns was used that offered optimum energy at 850 nm [56]. The optical illumination profile achieved with the two 850 nm LED arrays operated with pulse widths of 70 ns is shown in Figure 1g. The shape of the beam falling on skin is approximately a rectangle with an area of 9 cm^2^ (5 by 1.8 cm), leading to an incident optical fluence of 0.044 mJ/cm^2^, considering 400 μJ total energy per pulse with the two LED arrays. The pulse repetition frequency (PRF) of these LEDs can also be controlled in the range of 1 to 4 KHz allowing multiple averaging options leading to different frame rates. To assess the effect of changing the frame rate over the PA image quality, several different combinations of PRF and frame averaging are used in this study, as discussed in the subsequent sections. The US probe used in this study is a 128-element linear US array with a pitch of 0.3 mm, center frequency of 7 MHz, elevational focus of 15 mm and a measured 6 dB bandwidth of 75%. The system provides PA and US acquisition sampling rates of 40 and 20 MHz, respectively.

### 2.2. Experimental Setup for Comparing LED-PAUS and Laser-PAUS

Figure 1 presents the overall experimental setup developed to compare the performance of LED-PAUS and laser-PAUS imaging. The LED-PAUS system, described in Section 2.1, was adapted for laser-PAUS imaging. In laser-PAUS, a portable optical parametric oscillator (OPO) laser source (Phocus Mobile, Opotek, Inc., Carlsbad, CA, USA), tunable in the range of 690–950 nm, shown in Figure 1c, provided the laser illumination. The laser has a fixed pulse width of 5–7 ns, a fixed PRF of 10 Hz and an output energy of 140 mJ per pulse at 750 nm. For this study, 850 nm wavelength laser illumination was used with the optical energy tuned down to 40 mJ/pulse. Light output from the laser was coupled to a 2 m long custom designed optical fiber bundle (Fiberoptic System Inc., Simi Valley, CA, USA). The fiber had a fused end with a diameter of 6.5 mm that entered into the tunable output port of the laser, as shown in Figure 1c. The distal end of this fiber was split into twenty smaller (1.45 mm inner diameter) fibers with numerical apertures of 0.55, each sharing equal optical energy. Ten out of these twenty fibers were inserted into each of the two custom designed 3-D printed fiber holders attached each side of the US probe, as shown in Figure 1f. This design allowed laser illumination similar to the case of LED arrays, in terms of the illumination angle and the overall geometry around the US probe. Further, to achieve a uniform illumination profile on the tissue surface, two glass diffusers (N-BK7 Ground Glass Diffuser 1500 Grit, Thorlabs Inc., Newton, NJ, USA) were attached at the output end of the fiber holders, as shown in Figure 1f. The resulting laser-illumination profile at 850 nm is shown in Figure 1h. The shape of the beam falling on skin is approximately a rectangle with an area of 6 cm^2^ (5 by 1.2 cm), leading to an incident optical fluence of 6.66 mJ/cm^2^, considering 40 mJ total energy per pulse with the laser illumination. In our experimental setup, switching from LED arrays to a laser fiber setup and vice versa was convenient and did not disturb the US probe as well as the imaging subject. Figure 1i,j show the heads of LED-PAUS and laser-PAUS systems for acquiring respective B-mode PA and US images of human hand vasculature.

## 3. Validation Experiments and Results

In this section, an extensive evaluation of the imaging performance of low-power LED-PAUS and high-power laser-PAUS systems is presented with the help of rigorous SNR and resolution studies on several tissue phantoms, in vivo imaging of the human wrist and in vivo 3-D vasculature mapping of the human forearm.

### 3.1. Photoacoustic Imaging Comparison of LED Arrays and Laser Source Using a Scattering Phantom

**Figure 2:**
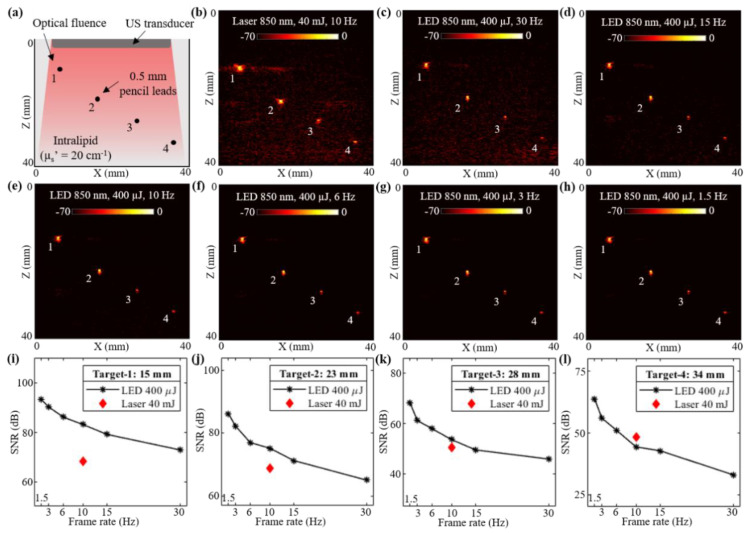
Performance evaluation of LED array-based and laser-illumination-based photoacoustic (PA) imaging in intralipid scattering phantom. (**a**) Shows the schematic of a scattering phantom with four 0.5 mm diameter pencil leads placed at 15, 23, 28 and 34 mm depth from the ultrasound (US) transducer inside intralipid medium mimicking an optical reduced scattering coefficient of 20 cm^−1^. (**b**) Shows the PA imaging results with 850 nm laser illumination at 10 Hz and 40 mJ optical energy with <20 mJ/cm^2^ optical fluence on the phantom surface. (**c**–**h**) Show the PA imaging results with LED array-based illumination at an 850 nm wavelength, a total of 400 μJ output energy from the two LED arrays and frame rates of 30, 15, 10, 6, 3 and 1.5 Hz, respectively. (**i**–**l**) Show the plots of signal-to-noise ratio (SNR) with respect to the acquisition frame rates comparing LED array and laser-illumination-based PA imaging performance for the pencil lead targets at four depths.

In this subsection, a controlled study evaluating the deep tissue PAI capabilities of low-power LED arrays and a high-power laser source is presented. An acrylic tank with four holes was fabricated and a pencil lead with a diameter of 0.5 mm was inserted into each hole. The tank was filled with an intralipid solution to mimic an optical reduced scattering coefficient (${µ}_{s}^{\prime }$) of 20 cm^−1^. Considering an approximate optical absorption coefficient (${µ}_{a}$) of 0.05 to 0.1 cm^−1^, the effective attenuation coefficient (${µ}_{eff}$) of the intralipid phantom was in the range of 1.73 to 2.45 cm^−1^ (${µ}_{eff}\;=\;\sqrt{3{µ}_{a}\left({µ}_{a}+{µ}_{s}^{\prime }\right)}$). Figure 2a shows the schematic of the experimental setup with four pencil lead targets diagonally arranged along the depth of imaging inside the intralipid solution. The measured depth of these four pencil leads from the surface of the US transducer were 15, 23, 28, and 34 mm, respectively.

The laser-illumination setup discussed in Section 2.2 was first employed to capture the PA images. The AcousticX data acquisition system software was synchronized to the laser acquisition mode. Two external triggers from the system were used for driving the laser. One trigger from the synch-1 port of the system went into the flash-lamp input port of the OPO laser and the other trigger from the synch-2 port fed the Q-Switch IN port of the laser, as shown in Figure 1b,c. After switching ON the 2-D B-mode RF data acquisition, the laser flash-lamp was first turned ON followed by (after 10s delay) the laser, from a PC connected to the laser. Once the laser was ON and synchronized with the data acquisition, PA data could be captured at a 10 Hz frame rate (limited by the PRF of the laser). Figure 2b shows a PA image captured with the laser illumination at 850 nm wavelength and 40 mJ/pulse optical energy resulting in <20 mJ/cm^2^ optical fluence. As shown, all four pencil lead targets generated PA signals due to their higher optical absorptions compared to the intralipid medium.

To acquire the PA images with LED arrays, the laser arrangement was removed and the two 850 nm wavelength LED arrays were attached to the US transducer as shown in Figure 1e. The software of the AcousticX system was set to the LED acquisition mode. The PRF for the LED arrays was selected as 4 KHz. Figure 2c shows a PA image captured using the LED array setup with a frame averaging of 128. The achieved frame rate in this configuration was 30 Hz (128 averaging at 4 KHz PRF). Further, to study the effect of frame averaging over the LED-PA imaging performance, the averaging was increased from 128 to 256, 384, 640, 1280 and 2560 frames, leading to frame rates of 15, 10, 6, 3, and 1.5 Hz, respectively. The corresponding PA images are shown in Figure 2d–h.

In order to perform an effective comparison of the captured PA images with the laser and the LED array illuminations, the raw PA data were extracted and analyzed in a local computer using MATLAB software. First, frequency domain reconstruction [57] was performed to beamform the PA images from the raw data. All beamformed PA images were then log-compressed, maintaining the same 70 dB scale, as presented in Figure 2b–h, for effective comparison. Further, to quantitatively compare these PA images, the SNR study was performed over all four pencil lead targets. For calculating the SNR, peak PA signal at the target locations (over a circular region surrounding the targets) and the mean noise adjacent to each target (over a similar circular region at same depth as targets) were calculated over the linear beamformed PA images. The calculated values of peak signal, mean noise and SNR for the four targets over the PA images corresponding to the varying frame averaging of laser acquisition (1, 2, 4, 10, 128 frame averages leading to 10 Hz, 5 Hz, 2.5 Hz, 1 Hz, and 78 mHz frame rates when computed offline) and the LED array acquisition at varying frame rates are presented in Table 1. The calculations at 78 mHz with the laser system were specifically performed in order to compare the SNR of two systems at the same frame averaging—i.e., 128 frames (LED 30 Hz). As shown, the peak PA signal for the laser acquisition is about two log orders higher than that for the LED arrays. However, the mean noise in the case of laser illumination is even higher (up to three log orders), leading to a lower SNR, especially when compared at shallow imaging depths.

Plots in Figure 2i–l present the SNR trends observed with varying frame rates of LED array acquisitions for the four targets. The corresponding value of SNR for the laser acquisition at 10 Hz is also marked in each of these plots. For the targets lower than 30 mm depth, the LED-based PAI at 10 Hz frame rate continued to show a higher SNR. Target-4 at 34 mm depth was detected at a higher SNR with the laser illumination as compared to the LED arrays at the same frame rate. However, increasing the PA frame averaging further for the LED arrays acquisition led to a lower frame rate (<10 Hz), and helped in boosting the SNR value for higher depth targets. Further, when maintaining same frame averaging (128 frames) for the laser (78 mHz frame rate) and the LED arrays (30 Hz frame rate), significantly high SNRs were observed for all four targets with the laser, sacrificing the real-time imaging.

In order to validate the optical properties of our phantom, we calculated the ${µ}_{eff}$ value from the experimental peak PA signal values for LED 10 Hz data. Using the Beer Lambert’s principle, i.e.,$\;I\left(z\right)=I_{o}e^{-{µ}_{eff}z}$, where *I(z)* is the PA intensity at depth (z) cm and *I_o_* is the intensity at zero depth, and taking the ratio of two equations at two different depths (z_1_ at 1.5 cm and z_2_ at 3.4 cm) will cancel out the *I_o_* and lead to the ${µ}_{eff}$ 2.034 cm^−1^. This closely matches to the previously reported values in the range of 1.734 to 2.456 cm^−1^, mentioned above in the phantom description.

### 3.2. Photoacoustic Imaging Comparision of LED Arrays and Laser Illuminations over Chicken Tissue Phantom

**Figure 3:**
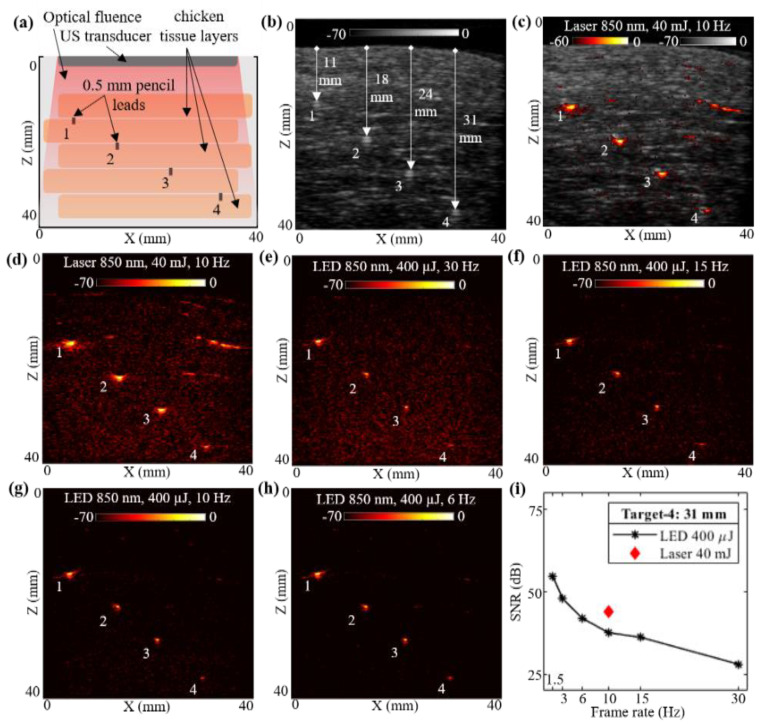
Performance evaluation of LED array-based and laser-based photoacoustic (PA) imaging in chicken tissue phantom. (**a**) Shows a schematic of chicken tissue phantom with five layers of chicken breast tissue stacked inside water tank. The positions of four 0.5 mm diameter pencil lead targets placed in between the chicken tissue layers are also shown. (**b**–**d**) Show the B-mode ultrasound (US), coregistered US + PA and PA image, respectively, captured with laser-based illumination at 850 nm wavelength and 10 Hz high pulse repetition frequency (PRF). (**e**–**h**) Show the PA imaging results obtained by imaging the chicken tissue phantom using LED array-based illumination, at 850 nm illumination and frame rates of 30, 15, 10, and 6 Hz, respectively. (**i**) Shows the plot of signal-to-noise ratio (SNR) with respect to the frame rate comparing the LED array and laser-illumination-based PA imaging performance for the deepest pencil lead target (target-4).

To compare the deep tissue PAI capabilities of the low-power LED arrays and the high-power laser illuminations, a multilayer chicken tissue phantom was designed. Figure 3a shows the schematic of the chicken tissue phantom with five layers of chicken breast tissue stacked inside a water tank, with an estimated optical absorption and reduced scattering coefficients of 0.1 to 0.2 cm^−1^ and 1.0 to 5 cm^−1^, respectively, with an 850 nm wavelength [58], leading to an effective attenuation coefficient (${µ}_{eff}$) in the range of 0.575 to 1.766 cm^−1^ (${µ}_{eff}\;=\;\sqrt{3{µ}_{a}\left({µ}_{a}+{µ}_{s}^{\prime }\right)}$). Four pencil leads with diameters of 0.5 mm were placed in between the chicken tissue layers as shown. The measured depths of these pencil lead targets from the top layer of the chicken tissue were 11, 18, 24, and 31 mm, respectively.

With the above-described phantom, a laser-illumination setup was used to acquire the US and PA images at 10 Hz, maintaining the same 40 mJ output energy (<20 mJ/cm^2^ optical fluence on the phantom surface) and laser with an 850 nm wavelength. The captured raw data from the AcousticX software were reconstructed in MATLAB to further perform the quantitative comparison. Figure 3b shows the beamformed B-mode US image of the chicken tissue phantom, at a log scale of 70 dB, clearly highlighting the chicken tissue structure. The US image also shows the distance of the four pencil lead targets from the top layer of chicken tissue. Similarly, the PA raw data captured from the software were reconstructed, log-compressed and was overlaid on the US image to generate a co-registered US + PA image of the phantom. Figure 3c shows the coregistered US and PA image at a 60 dB scale in order to keep the noise floor at the threshold. The beamformed PA image with 70 dB log scale is shown in Figure 3d.

Without disturbing the phantom, the PA images were subsequently acquired with the LED arrays setup. The laser arrangement was removed and the two 850 nm LED arrays were attached to the US probe. With the 4 KHz PRF of the LED arrays, the PA images were captured at varying frame averaging settings, similar to the settings used for Section 3.1, leading to the frame rates of 30, 15, 10, 6, 3 and 1.5 Hz. The captured raw data from the AcousticX software were again extracted in the MATLAB software and reconstructed to generate B-mode PA images for comparing with the laser PA images. All PA images were compressed at the 70 dB log scale for effective comparison. Four representative PA images at 30, 15, 10, and 6 Hz, respectively, are shown in Figure 3e–h.

Further, for a quantitative comparison of high-power laser versus low power LED array-based acquisitions over the chicken tissue phantom, the SNR study was performed over the linear beamformed images in MATLAB. For this experiment, we studied the SNR of the shallowest target (target-1 located 11 mm) and the deepest target (target-4 located 31 mm) deep inside chicken breast tissue. For calculating the SNR, the peak signal at target locations and the mean noise adjacent to these targets were calculated. The values of peak PA signal, mean adjacent noise and the SNR for laser-based and LED array-based acquisitions at varying frame rates are listed in Table 2. The peak signal as well as mean noise for laser acquisition at 10 Hz is about two to three log orders of magnitude higher compared to the LED arrays. However, the SNR for LEDs is still comparable to the lasers. Figure 3i also demonstrates the trend of SNR for target-4, comparing the laser and the LED array illuminations at varying frame rates. For a 31 mm deep target, LED arrays provide close to 37.51 dB SNRs, whereas laser illumination provides about 43.75 dB SNR at a 10 Hz frame rate. When increasing the frame averaging, hence a reduction in the frame rate, LED arrays show significant improvement of SNR for the same target, up to 54.47 dB at 1.5 Hz, which is higher than the SNR of laser illumination at 10 Hz. These results demonstrate the capabilities of LED arrays to image deeper inside realistic tissue medium and motivated us to further study how they compare with high-power laser sources for imaging in vivo human vasculature.

Similar to Section 3.1, we validated the optical properties of this chicken tissue phantom by calculating the ${µ}_{eff}$ value from the experimental peak PA signal values for LED 10 Hz data. The experimentally calculated ${µ}_{eff}$ (2.067 cm^−1^) is slightly higher than the previously calculated range mentioned above in the phantom description. The small discrepancy could be due to chicken tissue heterogeneity—prolonged imaging of chicken tissue in water medium that increases the optical attenuation.

### 3.3. Photoacoustic Imaging Comparison of LED Arrays and Laser Sources: Resolution Study

**Figure 4:**
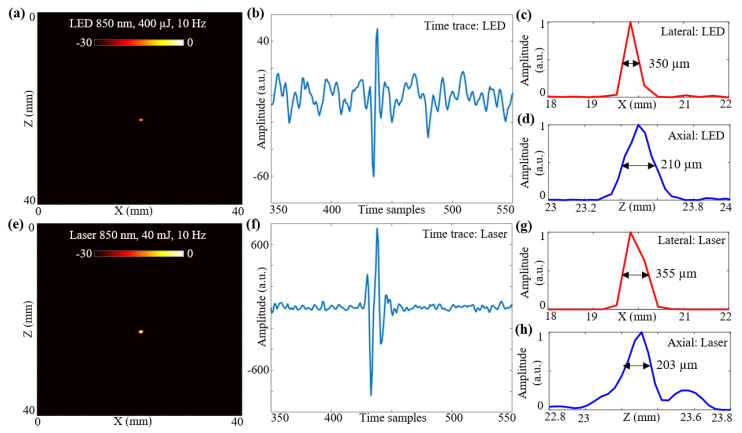
Resolution study for LED array-based and laser-illumination-based photoacoustic (PA) imaging. (**a**) B-mode PA image obtained for a 30 μm carbon fiber placed in an intralipid-based phantom using LED illumination at 850 nm with a frame rate of 10 Hz. (**b**) Shows zoomed time trace of an A-line across the target region for the PA data acquired with LED illumination. (**c**,**d**) Show the line-spread functions of the PA amplitudes for the carbon fiber target plotted in the lateral and axial directions, respectively, when imaged with LED setup. The obtained lateral and axial resolutions with the full-width-half-maximum (FWHM) approach are 350 and 210 μm, respectively. (**e**) B-mode PA image obtained for same carbon fiber phantom using laser illumination at 850 nm and 10 Hz frame rate. (**f**) Shows the time trace for the PA data acquired with laser illumination. (**g**,**h**) Show the line-spread functions of the PA amplitudes in lateral and axial directions with the laser illumination. The obtained lateral and axial resolutions using the FWHM approach are 355 and 203 μm, respectively.

In this subsection, the spatial resolutions of the two optical illumination setups, the LED-PAUS and the laser-PAUS, are characterized. A 30 μm carbon fiber was placed in a bath containing water mixed with intralipid to obtain a scattering medium of 3 cm^−1^. First, the two 850 nm LED arrays were attached to the US probe and a B-mode PA image of the phantom was acquired. The raw data captured were extracted in the MATLAB software and reconstructed to generate a B-mode image. The log-compressed B-mode PA image at a 30 dB scale is shown in Figure 4a. Figure 4b presents a sample zoomed time trace of an A-line across the target region for the PA data acquired with LED array illumination. To calculate the spatial resolution for this carbon fiber target, the line-spread functions of the PA amplitudes are plotted in the lateral and axial directions, respectively, as shown in Figure 4c,d. The obtained lateral and axial resolutions with a full-width-half-maximum (FWHM) approach are 350 and 210 μm, respectively.

To acquire the PA data with laser-illumination, the LED arrays were removed from the US probe and the laser fiber setup was attached without disturbing the phantom. The captured PA data were extracted and beamformed. Figure 4e shows the B-mode PA image, at a 30 dB scale, obtained for the same carbon fiber phantom using laser-illumination at 850 nm with 40 mJ output energy and a 10 Hz frame rate. A sample zoomed time trace of an A-line across the target region for the PA data acquired with the laser-illumination is shown in Figure 4f. The line-spread functions of the PA amplitudes in the lateral and axial directions with the laser illumination are shown in Figure 4g,h. The obtained lateral and axial resolutions using an FWHM approach for the laser illumination are 355 and 203 μm, respectively.

### 3.4. In Vivo Photoacoustic Imaging Comparison of LED Arrays and Laser Illumination over In Vivo Human Wrist

**Figure 5:**
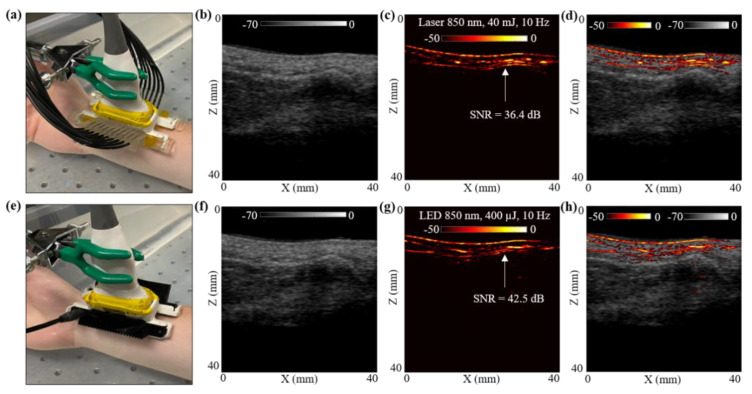
In vivo comparison of LED array-based and laser-based PA vasculature imaging over the right-hand wrist of a healthy 25-year-old male human volunteer. (**a**) Shows the experimental setup with the right-hand wrist placed inside a big water bath for the laser-based PA imaging. (**b**–**d**) Show the obtained US, PA and coregistered US + PA images for the setup shown in (**a**). (**e**) Shows the setup with LED arrays. (**f**–**h**) Show the obtained US, PA, and coregistered US + PA images for the setup shown in (**e**).

In this subsection, the photoacoustic vascular imaging capabilities of the LED array and the laser illumination were compared by in vivo imaging of a healthy human volunteer’s wrist vasculature. The volunteer was a healthy 25-year-old European male, and the experiment was conducted by following the internal imaging protocol of CYBERDYNE, INC (Rotterdam, The Netherlands) for healthy-volunteer imaging studies. For this study, the volunteer’s right hand was positioned inside a large water tank, as shown in Figure 5a. The probe was positioned such that one of the major blood vessels, which supplies blood to the forearm and hand, is within the field-of-view (FOV).

The hand was first imaged with the laser-PAUS setup by attaching the laser fiber holders to the US probe, as shown in Figure 5a. The laser was operated at 850 nm wavelength, 10 Hz PRF, and delivered output optical energy of 40 mJ. This allowed ANSI safety limits of <20 mJ/cm^2^ optical fluence on the hand surface [59]. During the real-time data acquisition, the probe was aligned such that the major blood vessel could be seen running parallel to the skin surface in the PA images. The captured US and PA raw data using the AcousticX software were later extracted in the MATLAB software and were reconstructed to generate the beamformed images. The beamformed, log-compressed B-mode US, PA and the coregistered US + PA images are shown in Figure 5b–d. The US image showed the anatomical features along the depth of human wrist, whereas the PA image highlighted the major blood vasculature. There was fairly strong correspondence between the locations of the blood vessel in the PA image and the appearance of anechoic regions in the US image. Based on the anatomy of the vasculature in human wrist, the PA signals ~5 mm below the skin surface may have corresponded to the radial artery that travels across the front of the elbow, deep under the muscle until it comes to the wrist where it comes close to the skin surface. This is also marked with a white arrow in the PA image in Figure 5c.

To compare these laser-illumination results of the human wrist with the LED arrays, the laser-fiber attachments were gently removed without disturbing the location of the US probe. The two 850 nm LED arrays were then attached to the US probe, as shown in Figure 5e. With a PRF of 4 KHz and frame averaging of 384, leading to a frame rate of 10 Hz, the US and PA frames were captured using the LED array setup. The US and PA raw data were then reconstructed in MATLAB. Figure 5f–h show the beamformed log-compressed B-mode US, PA and coregistered US + PA images for the human wrist. As in the case of laser illumination, the LED array-based PA images also imaged the same vasculature below the skin surface. The radial artery present ~5 mm below the skin was clearly visible with the LED array-based acquisition as well.

To further compare the two setups quantitatively, an SNR comparison study was performed for the radial artery, as marked with white arrows in Figure 5c,g. To calculate the SNR, the peak PA signal at the artery and the mean noise adjacent to the artery region was calculated over the linear beamformed PA images resulting from the laser-illumination and the LED array-based acquisitions. Table 3 presents the values of peak PA signal, mean noise and the SNR. The SNR values are also marked in the Figure 5c,g. Both the peak signal and mean noise with the laser are up to three log orders of magnitude higher compared to the LEDs. However, the SNR value for the laser-illumination-based PA image was about 6 dB lower than the SNR with the LED array acquisition. This follows the trend observed for the controlled tissue phantom studies discussed in Section 3.1, where the shallow targets (<30 mm) inside an intralipid medium were detected with higher SNRs using LED arrays compared to the laser illumination, maintaining the same frame rate.

### 3.5. In Vivo Photoacoustic Imaging Comparison of LED Arrays and Laser Illumination: In Vivo Human Forearm

**Figure 6:**
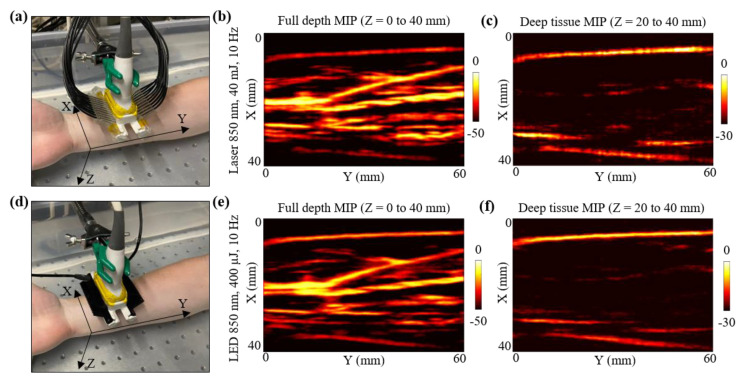
In vivo comparison of LED array-based and laser-based PA vasculature imaging over forearm of a healthy 25-year-old male human volunteer. (**a**) Shows the experimental setup with the right forearm placed inside big water bath for the laser-based PA imaging. (**b**,**c**) Show the obtained full depth and deep tissue maximum-intensity-projections (MIPs) of the reconstructed 3-D PA volume for the setup shown in (**a**). (**d**) Shows the setup with LED arrays. (**e**,**f**) Show the obtained full depth and deep tissue MIPs of the reconstructed 3-D PA volume for the setup shown in (**d**).

This subsection presents in vivo 3-D mapping of the vasculature inside a human volunteer’s forearm, using the LED array-based and the laser-based illuminations. For this study, the volunteer was a healthy 25-year-old European male, and the experiment was conducted by following the internal imaging protocol of CYBERDYNE, INC (Rotterdam, The Netherlands) for healthy-volunteer imaging experiments. The forearm of the volunteer was submerged in water. The US probe was fixed on a linear translation stage, translating in the Y-direction, as shown in Figure 6a.

For the laser acquisition, the laser-illumination setup was attached to the US probe. After switching ON the laser software, the RF data acquisition on the AcousticX software were first turned ON. Note that the 3-D data acquisition feature of the AcousticX software does not work for the laser mode. Therefore, to capture the data corresponding to the Y-direction scan, a manual stage motion feature was used while acquiring the data in the 2-D RF acquisition mode. The linear stage was translated for 60 mm in the Y-direction while capturing the RF data. The laser was tuned to 850 nm at 40 mJ output energy (<20 mJ/cm^2^ optical fluence on the hand surface) and 10 Hz PRF. The captured PA raw data were later extracted and reconstructed in MATLAB. Initial PA frames captured prior to the stage motion were discarded for the 3-D volumetric reconstruction. Figure 6b presents the maximum-intensity-projection (MIP) of the 3-D PA volume for full depth, highlighting the major vasculature present in the human forearm. To better visualize the vasculature deeper than ~5 mm, a deep tissue MIP of the PA volume is also shown in Figure 6c. Considering the initial 15 mm stand-off of the US probe from the human skin surface, the deep tissue MIP fell within Z = 20 to 40 mm, where Z is the depth dimension. Since deeper blood vessels show weak PA intensities, the MIP image in Figure 6c was scaled to 30 dB as opposed to the full depth MIP in Figure 6b, which was scaled to 50 dB.

After the laser acquisition, the stage was manually moved back to the home position and the laser setup was detached from the US probe without disturbing the position of the forearm with respect to the probe. To acquire the LED array-based PA 3-D scan data, two 850 nm LED arrays were attached to the US probe. In this case, the AcousticX software directly allows 3-D PA data acquisition using the automatic 3-D scan feature. The same Y-direction translation stage was automatically scanned for 60 mm and the raw data captured were extracted in MATLAB. The beamformed PA frames were stitched in the Y-dimension to reconstruct a 3-D PA volume. Figure 6d,e present the full depth MIP and the deep tissue MIP of the 3-D PA volume, respectively.

Qualitatively, Figure 6b,e look very similar, indicating that both laser and LED array-based illumination are able to map the major vasculature present inside human forearm. Minor differences in the SNR for deep vessels can be noticed when comparing the deep tissue MIPs presented in Figure 6c,f. For example, a deeper blood vessel running parallel to the *Y*-axis at around X = 20 mm (in Figure 6c,f) was detected with a better SNR with the laser source as compared to the LED arrays. Similarly, the deep tissue MIP from laser acquisition show other minor PA signals (at coordinates X,Y = 30 mm, 50 mm), that are relatively weaker in the deep tissue MIP from the LED acquisitions, emphasizing the need for a high-power laser when imaging very deep (>2 cm) vasculature.

## 4. Discussion

To successfully translate PAI to the POC applications and to resource-limited settings, the cost and the overall size of the PAI systems need to be significantly cut down. LED arrays are one of the highly explored optical sources in the PA literature that are portable and significantly lower in cost and footprint. This study was focused on comparing the capabilities of these LED arrays (approximately USD 15K, including driver electronics) with a state-of-the-art high-power OPO laser (approximately USD 100K) for PAI. A commercial LED-PAUS system, AcousticX, was first adapted to perform sequential acquisitions of LED-PAUS and laser-PAUS. This ensured that the PA and US signals were detected by the same ultrasound transducer array and processed by the same data acquisition system. To further draw a fair comparison, the experimental setup shown in Figure 1 allowed for (1) similar optical illumination on the tissue surface in terms of the geometry, angle and aperture of illumination, and (2) a convenient, uninterrupted sequential acquisition of PA images with the two sources. To achieve uniform laser illumination on the tissue surface, two glass diffusers were attached to the output end of the laser fiber holders. For all the experimental studies presented in this work, the output optical energy for the laser and the LED arrays were maintained at 40 mJ and 400 μJ/pulse, respectively, with an 850 nm wavelength of illumination. A constant PRF of 10 Hz for the laser and 4 KHz for the LED arrays were used for all studies. With no frame averaging, the obtained PA frame rate for laser was 10 Hz. For the LED arrays, the PA frame averaging was varied from 128 to 256, 384, 640, 1280, and 2560, achieving the frame rates of 30, 15, 10, 6, 3, and 1.5 Hz, respectively.

The first comparison study presented in Section 3.1 consisted of controlled experiments on an intralipid phantom shown in Figure 2. The four 0.5 mm pencil lead targets arranged diagonally along the depth of the scattering phantom were imaged using both laser and LED arrays. Detailed analysis of the SNR presented in Table 1 and plotted in Figure 2i–l led to the following conclusions on the quantitative performance of the two sources. (1) At similar frame rates (10 Hz), LED-PAUS imaging showed higher SNR compared to laser-PAUS imaging for targets up to an imaging depth of 30 mm. (2) Laser-PAUS imaging provided a better SNR for the deeper targets (>30 mm) as compared to the LED-PAUS at 10 Hz. (3) With increased frame averaging and hence a reduced overall frame rate (<10 Hz), the deep tissue (>30 mm) performance of LED arrays can be improved. For example, the target-4 located 34 mm deep was imaged with a 48.24 dB SNR using laser illumination and with 44.22 dB SNR using LED arrays, both at 10 Hz. However, with increase in PA averaging (lower frame rates), LED arrays were able to provide an improved SNR of up to 50.92, 55.79, and 63.55 dB, when imaged at 6, 3, and 1.5 Hz frame rates, respectively. Further, comparison of the magnitudes of peak PA signals from the target and mean noise surrounding the target for the two optical illuminations led to the following observations. While higher optical energy from the laser source generates a PA signal two to three log orders of magnitude higher compared to the lower-power LED arrays, the average noise floor from the background regions was also observed to be up to three to four log orders higher for the laser sources compared to LED arrays. In conclusion, the low-power LED arrays were effective due to lower noise floor that comes from the higher frame averaging possible with high PRF (4 KHz) of LED arrays.

Our next comparison study, presented in Section 3.2, involved a multilayer chicken tissue phantom with four 0.5 mm pencil lead targets embedded in between the five layers of chicken breast tissue. Figure 3 presented the qualitative comparison of laser-PAUS and LED-PAUS, whereas Table 2 highlighted the quantitative comparison of PAI performance for the deepest pencil lead target—i.e., target-4 at 31 mm. This study results further confirmed our observations with the intralipid scattering phantom presented in Section 3.1. For example: (1) both peak PA signal and mean noise from the laser illumination were up to three log orders of magnitude higher compared to the LED array illumination. (2) For the target-4 at 31 mm, the laser showed a ~6 dB higher SNR compared to the LED arrays at a 10 Hz PA frame rate. (3) With the increase in the average, and hence reduced, frame rate (<10 Hz), LED arrays show higher SNR values than laser illumination, obtaining SNRs of 54.47 and 47.76 dB at 1.5 and 3 Hz, respectively, for the same target-4 as compared to an SNR of 43.75 dB with the laser.

The study presented in Section 3.3 compared the lateral and axial resolutions of the two setups, LED-PAUS and laser-PAUS, by imaging carbon fiber with a 30 μm diameter inside an intralipid-based phantom. Although the pulse widths for the laser (5–7 ns) and the LED arrays (70 ns) in this study were not similar, the spatial resolution were shown to be in the same ranges—i.e., ~350 μm lateral and ~205 μm axial. This confirmed the fact that the spatial resolution here is mainly limited by the acoustic detection.

In the final study, in Section 3.4 we presented an in vivo LED-PAUS and laser-PAUS imaging of the wrist of a healthy human volunteer. Figure 5 presents the qualitative and quantitative comparison results for a radial artery seen ~5 mm below the wrist skin surface. Analysis of the peak PA signal, mean noise and SNR for this radial artery is presented in Table 3. As observed with the studies presented in previous sections, for a shallow depth target, the LED-PAUS showed better SNR than the laser-PAUS at the same 10 Hz frame rates. The ~5 mm deep radial artery was imaged with a 36.37 dB SNR with the laser compared to a 42.49 dB SNR with LED arrays. This study substantiates that the LED-PAUS imaging is an attractive choice for several preclinical and clinical applications.

Further, in Section 3.5, to test the capabilities of deep tissue in vivo vascular imaging, we scanned the right-hand forearm of a healthy human volunteer. The 3-D vasculature map, presented in Figure 6, demonstrated that the LED arrays could image all the vessels that could be seen with a laser source, especially at shallow depths of up to 1.5 cm. For deeper (>30 mm) vessels, laser sources provided better signals. However, the amount of noise observed with the laser at those depths was also significantly high and thus limited the imaging performance when viewing deeper vessels. With an increased frame averaging with the LED arrays, the SNR of deeper vessels can potentially be improved.

The conclusive observations made by studying the performance of the two optical sources bring us to the following remarks. (1) LED arrays exhibit a strong potential for translating PAI systems to the resource-limited settings. (2) Higher frame averaging enhances the LED-PAUS imaging performance, especially for deeper targets (>30 mm), making it suitable for deep tissue imaging; however, this sacrifices the frame rate. To further improve the performance of LED-PAUS imaging, the state-of-the-art machine learning approaches can be employed that can help in (1) further boosting the frame rates by avoiding the need for higher frame averaging [60,61], and (2) improving the SNR for deep tissue targets [61,62].

## 5. Conclusions

Photoacoustic imaging capabilities of low-cost and low-power LED arrays were compared head-to-head with a high-power laser source using both tissue-mimicking phantoms and in vivo human subjects. The experimental observations on different tissue mimicking phantom studies demonstrated (1) high PRFs of LED arrays can be leveraged for averaging PA frames to achieve high SNR up to an imaging depth of 30 mm with 10 Hz frame rate, (2) high-power laser sources show higher SNRs for deeper targets (>30 mm) compared to the LED arrays at 10 Hz and (3) with increased frame averaging (<10 Hz), the SNR of a target at 34 mm depth with LED-PAUS closely matched that of laser-PAUS. The in vivo human hand vasculature imaging studies presented in this work also demonstrated similar observations. (1) The ~5 mm deep radial artery was imaged with a higher SNR using LED arrays in comparison to the laser source, and (2) for deeper vessels in the subject’s forearm, the laser at the 10 Hz frame rate had a slightly higher SNR compared to the LED arrays at 10 Hz. In summary, due to the low power of LED arrays, a higher frame averaging is required to image deep tissue targets. LED-PAUS holds strong potential in point-of-care diagnosis of vascular diseases.

## Figures and Tables

**Table 1:** Peak PA signal, average background noise and signal-to-noise ratio (SNR) values for four pencil lead targets located 15, 23, 28 and 34 mm deep inside scattering phantom imaged with LED array and laser illuminations at varying frame rates.

Configuration	Target-1: 15 mm			Target-2: 23 mm			Target-3: 28 mm			Target-4: 34 mm		
	Signal (a.u.)	Noise (a.u.)	SNR (dB)	Signal (a.u.)	Noise (a.u.)	SNR (dB)	Signal (a.u.)	Noise (a.u.)	SNR (dB)	Signal (a.u.)	Noise (a.u.)	SNR (dB)
**Laser 10 Hz**	2.62E14	1.04E11	68.01	4.09E14	1.49E11	68.77	7.96E13	2.38E11	50.48	4.19E13	1.62E11	48.24
**Laser 5 Hz**	2.60E14	5.01E10	74.31	4.25E14	7.39E10	75.18	8.58E13	1.43E11	55.55	4.38E13	8.81E10	53.93
**Laser 2.5 Hz**	2.58E14	2.94E10	78.85	4.07E14	4.26E10	79.59	8.17E13	8.26E10	59.90	3.8E13	4.01E10	59.55
**Laser 1 Hz**	2.58E14	1.44E10	85.05	4.05E14	2.25E10	85.10	7.89E13	6.64E10	61.50	3.79E13	1.66E10	67.18
**Laser 78 mHz**	2.47E14	5.66E09	92.79	3.39E14	7.95E09	92.58	5.94E13	3.86E10	63.80	2.89E13	3.35E09	78.50
**LED 1.5 Hz**	3.62E12	8.06E07	93.06	1.95E12	9.59E07	86.17	2.06E11	8.16E07	68.04	8.59E10	5.70E07	63.55
**LED 3.0 Hz**	3.57E12	1.11E08	90.14	1.94E12	1.50E08	82.33	1.84E11	1.62E08	61.13	7.80E10	1.27E08	55.79
**LED 6.0 Hz**	3.69E12	1.84E08	86.01	1.89E12	2.65E08	77.04	2.14E11	2.72E08	57.90	8.39E10	2.39E08	50.92
**LED 10 Hz**	3.66E12	2.57E08	83.06	2.06E12	3.55E08	75.26	1.95E11	4.08E08	53.58	7.64E10	4.70E08	44.22
**LED 15 Hz**	3.56E12	3.98E08	79.03	1.86E12	5.06E08	71.31	2.07E11	6.98E08	49.45	8.36E10	6.18E08	42.62
**LED 30 Hz**	3.70E12	8.49E08	72.79	1.95E12	1.08E09	65.12	2.38E11	1.21E09	45.84	7.22E10	1.63E09	32.92

**Table 2:** Peak PA signal, average background noise and signal-to-noise ratio (SNR) values for pencil lead targets located 11 and 31 mm deep inside chicken breast tissue imaged with LED array and laser illuminations at varying frame rates.

Configuration	Target-1: 11 mm			Target-4: 31 mm		
	Signal (a.u.)	Noise (a.u.)	SNR (dB)	Signal (a.u.)	Noise (a.u.)	SNR (dB)
**Laser 10 Hz**	1.4E14	1.2E11	61.63	2.1E12	1.4E11	43.75
**LED 1.5 Hz**	2.1E12	6.8E07	89.71	3.5E10	6.6E07	54.47
**LED 3.0 Hz**	2.1E12	9.2E08	87.35	3.4E10	1.4E08	47.76
**LED 6.0 Hz**	2.1E12	1.6E08	82.47	2.8E10	2.2E08	41.89
**LED 10 Hz**	2.0E12	2.8E08	77.13	3.2E10	4.3E08	37.51
**LED 15 Hz**	2.1E12	3.7E08	75.03	3.6E10	5.7E08	36.08
**LED 30 Hz**	2.1E12	7.4E08	69.06	2.9E10	1.2E09	27.98

**Table 3:** Peak PA signal, average background noise and signal-to-noise ratio (SNR) values for blood vessel target located ~5 mm below the skin surface of right-hand wrist of a healthy 25-year-old male human volunteer imaged with LED array and laser illumination at 10 Hz frame rate.

Configuration	Target: Blood Vessel ~5 mm below Skin Surface of Human Wrist		
	Signal (a.u.)	Noise (a.u.)	SNR (dB)
**Laser 10 Hz**	3.7E13	5.7E11	36.37
**LED 10 Hz**	5.3E10	4.0E08	42.49

## Data Availability

Data available on request from the authors.

## Abbreviations

PA	Photoacoustic
US	Ultrasound
PAI	Photoacoustic imaging
LED	Light-emitting diode
SNR	Signal to noise ratio
PRF	Pulse repetition frequency
OPO	Optical parametric oscillator
FWHM	Full width half maximum
MIP	Maximum intensity projection
